# Case report: Pathological differences in pulmonary arterial hypertension in long-term responders to calcium channel blockers

**DOI:** 10.3389/fcvm.2023.1295718

**Published:** 2023-11-02

**Authors:** Yuichi Tamura, Sayamaa Lkhagvadorj, Yudai Tamura, Asuka Furukawa, Shinsuke Aida, Hirotoshi Ebinuma, Takayuki Shiomi

**Affiliations:** ^1^ Pulmonary Hypertension Center, International University of Health and Welfare Mita Hospital, Tokyo, Japan; ^2^ Department of Cardiology, International University of Health and Welfare, School of Medicine, Chiba, Japan; ^3^ Department of Pathology, International University of Health and Welfare, School of Medicine, Chiba, Japan; ^4^ Department of Pathology, International University of Health and Welfare Mita Hospital, Tokyo, Japan; ^5^ Department of Gastroenterology and Hepatology, International University of Health and Welfare, School of Medicine, Chiba, Japan

**Keywords:** pulmonary arterial hypertension, calcium channel blockers, long term survivor, pathophysiology, pathology

## Abstract

**Background:**

This study investigates the pulmonary arterial histopathology in patients with idiopathic pulmonary arterial hypertension (IPAH) and acute vasoreactive phenotype, who demonstrated long-term survival (>30 years) and incidental death from causes other than PAH progression. The pathological changes observed in these patients were compared with those in patients with bone morphogenetic protein receptor type 2 (BMPR2) mutation.

**Case Presentation:**

We present two cases of patients with pulmonary arterial hypertension (PAH) who died incidentally from causes unrelated to PAH progression. We report compares pulmonary arterial histopathology in long-term survivors of CCB-responsive PAH patient and a hereditary PAH patient with a BMPR2 mutation. Lung specimens were analyzed using the Heath and Edwards (HE) classification and percentage muscular wall thickness (%MWT) of pulmonary arterioles. A significant difference in the severity of grading (*p* = 0.0001) and distribution between grades 1-2, 4 (*p* = 0.001), and 5 (*p* = 0.014) was observed between both patients. These findings suggest differential vascular pathology between the two cases, with CCB responders displaying more mild illness lesions compared to BMPR2 mutant patients.

**Conclusion:**

The study revealed that CCB responders exhibit more mild illness vascular lesions than BMPR2 mutant patients despite their long-term survival, suggesting a difference in vascular pathology between the two phenotypes.

## 1. Introduction

Acute vasoreactive phenotype has a prevalence of 12.5% among patients with idiopathic pulmonary arterial hypertension (IPAH), with 6.8% of patients showing long-term improvement with calcium channel blockers (CCBs); patients who respond to CCBs are reported to have long-term survival, with an average 7-year survival rate of 97% under favorable hemodynamics and mild pathologic vascular degeneration in their pulmonary arteries (1). There has been a lack of information on vascular pathology in cases of ultra-long-term survivors of more than 30 years.

This study evaluated the pathological changes in the pulmonary arteries of patients with stable pulmonary arterial hypertension (PAH) who incidentally died from causes other than PAH progression 34 years after onset. Compared to the pulmonary histopathology of PAH patients with bone morphogenetic protein receptor type 2 (BMPR2) mutation who also survived for a long time and died incidentally, most pathological manifestations in CCB responders reported in this study were mild illness lesions, even in cases with a very long survival period.

## 2. Case presentation

Case 1 was a 64-year-old woman. She was diagnosed with IPAH at 30 years of age following pregnancy. Her initial hemodynamic evaluation revealed that she had presented a 35-mmHg mPAP, 3.90-L/min CO, 7.18 Wood's-Units PVR, and presented responsiveness to CCB. Therefore, she was managed with high-dose CCB monotherapy for the first 20 years. Sequential additions of an endothelin receptor antagonist and a PDE-5 inhibitor to improve PH management stabilized her hemodynamic status in the last 14 years. The last hemodynamic evaluation was performed at 64 years of age and showed a 37-mmHg mPAP, 6.08-L/min CO, 3.62 Wood's-Units PVR, and NYHA Class II. The patient subsequently died at 64 years of age due to complications of infective endocarditis and sepsis triggered by a urinary tract infection, but she had never been hospitalized for right heart failure 34 years since PAH onset.

Case 2 was a 58-year-old man who was diagnosed with hereditary PAH associated with a BMPR2 nonsense-mutation at 46 years of age. He was treated with triple combination therapy, including continuous intravenous epoprostenol. His last hemodynamic evaluation was performed at 56 years of age, with a 53-mmHg mPAP, 4.37-L/min CO, 10.5 Wood's-Units PVR, and NYHA Class III. There were no further worsening PAH events, including right heart failure, but at the age of 57 years, he was diagnosed with colorectal cancer with multiple inoperable metastases and subsequently died at 58 years of age.

The lung specimens of two patients were fixed in formalin from both lungs during autopsy. Twelve tissue sections from each patient were embedded in paraffin, sectioned (each section was 3-µm thick), and stained with hematoxylin & eosin and elastin van gienson stain to determine the detailed morphologic structures. Pulmonary arterial and arteriole characteristics were examined under NanoZoomer NDP views 2 software (Hamamatsu, Tokyo, Japan) and graded from 1 to 6 according to the histologic Heath and Edwards (HE) classification (2).

The degree of pulmonary arterioles musculization was determined by measuring percentage muscular wall thickness (%MWT). The %MWT of small pulmonary arterioles between 20 and 50 µm in diameter was calculated as follows: %MWT = (2 × MWT/external diameter) ×100 (3). MWT was measured in the same direction as the external diameter on both sides of circular arterioles with a constant circumferential wall thickness, and two measurements were averaged to obtain the final value.3 Statistical analysis was performed using PASW version 21.0 (SPSS Inc., Chicago, IL, USA). Categorical variables are expressed as a frequency or percentage, the Chi-squared test was used for comparisons, and the Mann-Whitney post-hoc test was used to determine individual grade differences between patients. For continuous variables, which are expressed as mean ± standard deviation and were compared using a non-parametric Student's *t*-test, a *p*-value <0.05 was considered statistically significant.

**Figure 1:**
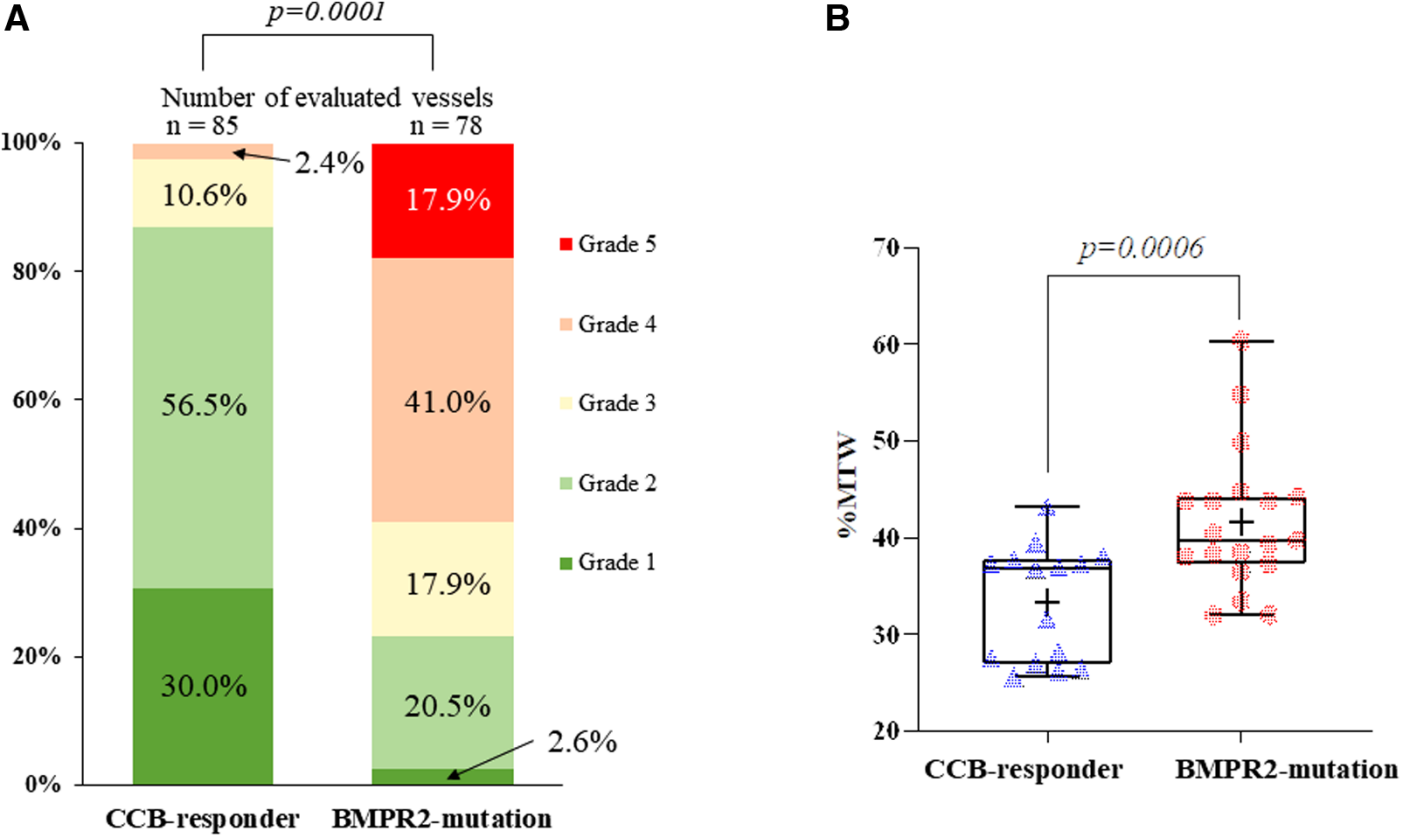
Comparison of histomorphological measurements. (**A**) According to the Heath and Edwards classification, the corresponding percentage for each grade (grades 1–6) in two cases is shown. No vascular changes were assessed as grade 6. There was a statistically significant difference in the severity and distribution of grading between cases (*p* = 0.0001). (**B**) The Box-and-Whisker diagram indicates the comparison of the percentage media wall thickness (%MWT) between case, with statistically significant differences (*p* = 0.0006). The box shows the interquartile range, with the median value indicated by the horizontal line; the mean value is indicated by (+); symbols indicate outliers.

**Figure 2:**
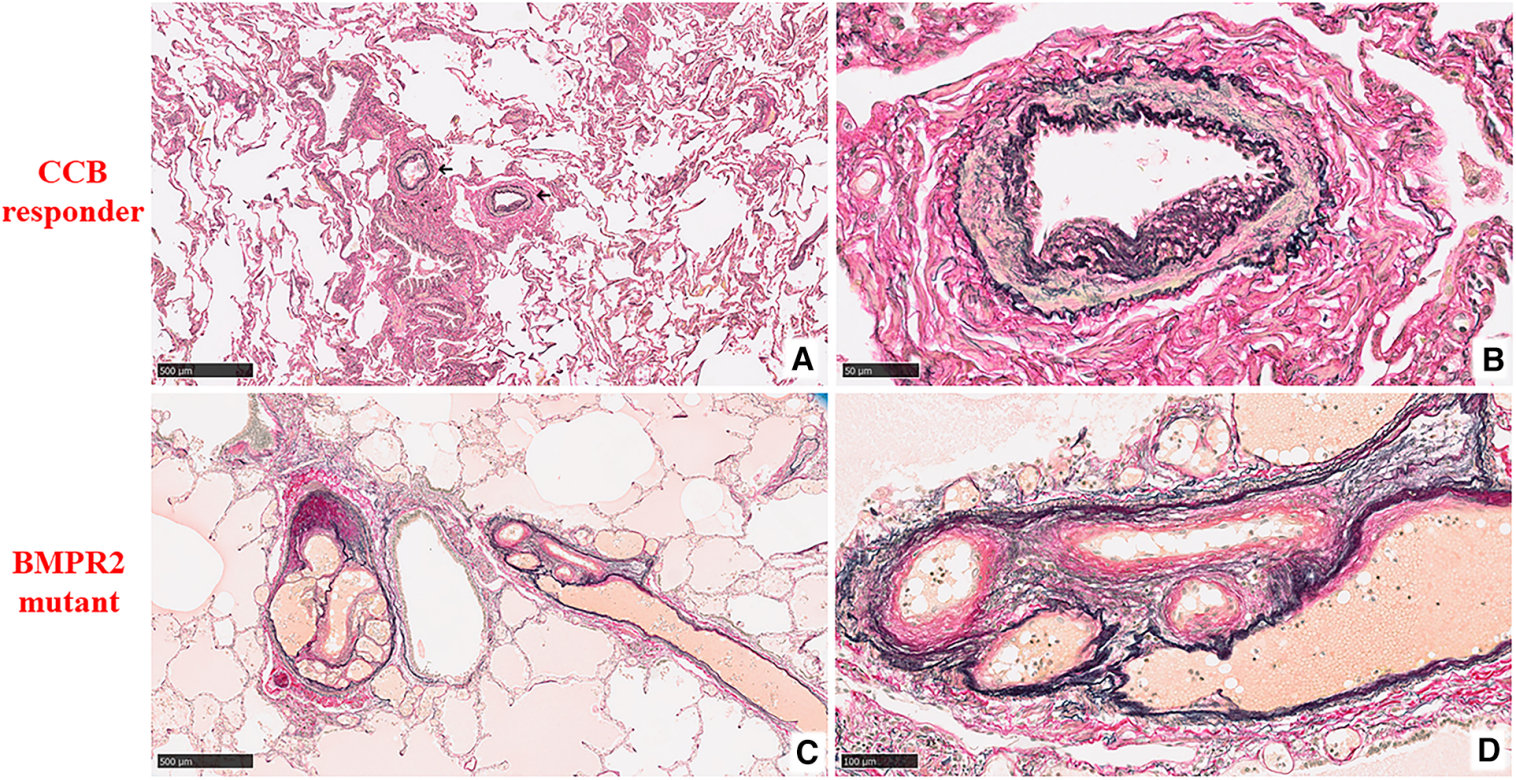
Histopathological findings of pulmonary arteries and arterioles by the heath and edwards classification (elastin van gienson stain). (**A**) At low magnification, two muscular arteries surrounding the bronchial tree are seen (marked with a black arrow) in the CCB responder. The remaining lung parenchyma is unremarkable (5×). (**B**) At high magnification, those pulmonary muscular arteries show medial hypertrophy with intimal cellular proliferation, which is classified as grade 2 (40×). (**C**) At low magnification, two muscular arteries on the right and left of the bronchiole are dilated and form an angiomatoid lesion in the BMPR2 mutant (5×). (**D**) At higher magnification, an oblique section of these formations is composed of several thin blood vascular structures having a wall of simple elastic tissue and medial and intimal fibroses, which are classified as grade 5 (20×).

According to the histologic HE classification, a statistically significant difference in the severity of grading (*p* = 0.0001; Figure 1A) and a distribution difference between grades 1-2, 4 (*p* = 0.001), and 5 (*p* = 0.014) were observed between both patients. In the CCB responder, the vessels presented mostly mild illness changes and a predominance of grade 2, showing medial hypertrophy (Figures 2A,B). However, in the BMPR 2 mutant case, an irreversible grade 4 was predominant, along with a worsening grade 5 (Figures 2C,D).

Small arterioles (20–50 μm in diameter) were measured, with an average vessel number of 17 ± 2. The mean external diameter was 39.6 ± 8.07 and 32.7 ± 7.5 µm in patients 1 and 2, respectively, and mean medial wall thickness was 6.5 ± 1.7 and 6.7 ± 1.8 µm. %MWT in the BMPR2 mutant (41.6% ± 7.3%) was significantly higher (*p* = 0.0006) than in the CCB responder (33.3% ± 5.9%) (Figure 1B).

## 3. Discussion

From the contrastive analysis of these two cases, we demonstrated that most pulmonary arteries in CCB responders with IPAH remain mild illness lesions with an HE classification of grade ≤3, regardless of whether the duration of IPAH is more than 30 years. In contrast, patients with BMPR2-positive IPAH show more irreversible lesions with an HE classification of grade 4–5, despite not reaching the end-stage of PH.

Hemins et al. have shown that the pathophysiology of CCB responders is related to Wnt/β-catenin signaling and intercellular adhesion based on their examination of RNA expression patterns (4). This suggests that pathophysiological differences exist between responder and nonresponder IPAH, which is characterized by BMP signaling abnormalities, resulting in vascular endothelial cell damage and vascular smooth muscle proliferation (5).

It has long been expected that the main lesions in CCB responder patients are localized in the smooth muscle cell layer of pre-capillaries since the administration of vasodilating agents rapidly decreases pulmonary vascular resistance. Pathological reports in young CCB responder patients have also shown only the medial hypertrophy of pulmonary arteries (6). Additionally, the only systematic pathology report suggested that CCB-responding PAH is characterized by a decreased number of vascular endothelial degenerations compared to non-CCB responding PAH (7). The present report illustrates the different long-term pathological characteristics of CCB responders, suggesting that different background mechanisms prevent irreversible degeneration of pulmonary vessels over a long period of time (≥30 years). Therefore, it can be expected that CCB responder patients can avoid irreversible changes in pulmonary arteries for a long period of time through continued appropriate treatment by not activating the remodeling signal.

## Data Availability

The raw data supporting the conclusions of this article will be made available by the authors, without undue reservation.
